# Roles of Enteric Neural Stem Cell Niche and Enteric Nervous System Development in Hirschsprung Disease

**DOI:** 10.3390/ijms22189659

**Published:** 2021-09-07

**Authors:** Yue Ji, Paul Kwong-Hang Tam, Clara Sze-Man Tang

**Affiliations:** 1 Li Dak-Sum Research Centre, The University of Hong Kong—Karolinska Institutet Collaboration in Regenerative Medicine, Hong Kong, China; jiyue@connect.hku.hk; 2 Department of Surgery, Li Ka Shing Faculty of Medicine, The University of Hong Kong, Hong Kong, China

**Keywords:** enteric nervous system, enteric neural crest cells, neural stem cell niche, extra-cellular matrix, Hirschsprung disease, regenerative medicine

## Abstract

The development of the enteric nervous system (ENS) is highly modulated by the synchronized interaction between the enteric neural crest cells (ENCCs) and the neural stem cell niche comprising the gut microenvironment. Genetic defects dysregulating the cellular behaviour(s) of the ENCCs result in incomplete innervation and hence ENS dysfunction. Hirschsprung disease (HSCR) is a rare complex neurocristopathy in which the enteric neural crest-derived cells fail to colonize the distal colon. In addition to ENS defects, increasing evidence suggests that HSCR patients may have intrinsic defects in the niche impairing the extracellular matrix (ECM)-cell interaction and/or dysregulating the cellular niche factors necessary for controlling stem cell behaviour. The niche defects in patients may compromise the regenerative capacity of the stem cell-based therapy and advocate for drug- and niche-based therapies as complementary therapeutic strategies to alleviate/enhance niche-cell interaction. Here, we provide a summary of the current understandings of the role of the enteric neural stem cell niche in modulating the development of the ENS and in the pathogenesis of HSCR. Deciphering the contribution of the niche to HSCR may provide important implications to the development of regenerative medicine for HSCR.

## 1. Introduction

The ENS is a major division of the autonomic nervous system [1]. It is a complex network of interconnected neurons and glia arranged in the myenteric and submucosal plexuses within the gut wall. ENS is known as the “second brain” for its extent, diversity, and complexity in neuronal cell types, as well as the importance in regulating autonomous intestinal functions. Sometimes it is also referred to the “first brain” as the development of the ENS evolutionarily precedes that of the central nervous system [2]. Majority of the ENS is derived from the vagal neural crest cells (NCCs) which migrate rostro-caudally from the neural tube to the foregut, proliferate, and differentiate into neurons and glia to colonize the whole intestine [3]. The sacral NCCs, originating from the second, caudal region of the neuraxis, contribute in a smaller extent to the ENS, mainly at the hindgut. These NCCs are originally premigratory and multipotent and are not programmed intrinsically to populate the entire bowel. Rather, the ENS develops in concert with the specialized gut microenvironment, known as the enteric neural stem cell “niche”, along which the NCCs migrate during embryogenesis. Through the spatiotemporal sensor–effector coupling, the neural stem cell niche orchestrates ENS development by modulating the migration, proliferation, survival, and differentiation of the ENS precursors. Defects disrupting one or more of these key processes or impairing the interaction with the stem cell niche may lead to incomplete innervation and thereby result in neurocristopathy of the ENS.

Hirschsprung disease (HSCR) is the most common enteric neurocristopathy and is the leading cause of functional intestinal obstruction in newborn babies. Although surgical resection is currently the mainstay of treatment, recent scientific breakthroughs in stem cell biology and tissue regeneration have opened a new avenue in regenerative therapies for HSCR. Growing evidence suggests that the interplay between the ENS precursors and the neural stem cell niche underlies the etiology of HSCR. In this review, we aim to summarize the recent findings on the association of the stem cell niche with disease pathogenesis and the emerging potential of the niche factors in regenerative medicine for HSCR. We also provide references to understand the coordinated interaction between the ENS precursors and the niche during ENS development from in vitro and in vivo studies. Better understanding of the bidirectional crosstalk between the ENS and the niche has important implications to devise novel regenerative therapeutic approaches not only for HSCR, but also for other neurogastrointestinal diseases.

## 2. Hirschsprung Disease and the Emerging Regenerative Therapeutic Options

HSCR, also known as congenital aganglionosis, is a complex neurocristopathy of the ENS. HSCR has high phenotypic variability and variable expressivity. Depending on the extent of aganglionosis, HSCR is classified into short segment HSCR (S-HSCR; 80%), long segment HSCR (L-HSCR; 15%), and total colonic aganglionosis (TCA; 5%) [4]. HSCR is generally believed to be caused by genetic defect(s) dysregulating the migration/proliferation/differentiation of the enteric NCCs (ENCCs) [5,6]. Surgical resection of the aganglionic bowel and anastomosis of the upstream ‘normo-ganglionic’ segment to the anus is currently the only treatment; however, it is not curative, as functional outcome is variable, and dysfunction, including gut dysmotility and enterocolitis, persists in many patients [7,8]. It is therefore imperative to develop novel non-surgical therapies to cure the lifelong sequelae and improve the quality of life of the patients.

Over the past decades, increasing emphasis has been placed on the development of stem cell-based therapy as a new treatment paradigm for HSCR [9,10]. Stem cell-based therapy involves the transplantation of allogeneic or autologous enteric neuronal stem/progenitor cells into the gut wall to reinnervate the aganglionic colon. These two approaches differ in the cellular sources for re-establishing a functional ENS and face different hurdles that needed to be overcome before clinical application. The allogeneic approach includes establishing ENS progenitors from embryonic stem cells derived from blastocysts or induced pluripotent stem cells (iPSCs) reprogrammed from healthy donor’s fibroblasts. Such approach may need to break the immunological barriers related to recipient rejection and necessitates special care in the differentiation of pluripotent stem cells into ENS lineage. The autologous strategy involves the use of (i) native ENCCs isolated from normoganglionic postnatal bowel of the patients, or (ii) ENS progenitors derived from patient-specific iPSCs. Transplantation of the autologous ENCCs has minimal risk of immune rejection, albeit with low quantity, poorer proliferation, and differentiation capacities partly attributed to the underlying genetic defects. Identification and additional correction of the underlying genetic culprit(s) using CRISPR/Cas9-mediated gene editing maybe needed to optimize the efficacy [11]. Encouragingly, multiple studies have shown that successful engraftment and functional integration of human enteric neural stem cells into the normal and aganglionic gut of wild type and diseased murine models, respectively can be achieved after transplantation, offering new hope to alternative therapies for HSCR [12,13,14].

While the functional integration of the grafted cells into the host gut is highly dependent on the interaction with the surrounding niche, little attention has been placed on whether the aganglionic bowel of patient with HSCR has intrinsic niche defect(s) interfering with the postnatal gut microenvironment. Such niche defects may compromise the regenerative capacity of the stem cell therapy and restoring the normal niche may promote the regenerative success. To date, the essential niche factors required for the ENS development remain largely uncharacterized and the contribution of cell-ECM interaction on disease development remains elusive. Deciphering the intrinsic niche factors impaired by genetic mutations in HSCR patients and the defective cell-ECM interaction would allow for the optimization of (i) in vitro niche condition for the growth and expansion of enteric neural stem cells in culture, as well as (ii) in vivo niche microenvironment to promote engraftment of exogenous ENS progenitors.

## 3. Known and Novel Contribution of Cellular Niche Factors to ENS Defect in HSCR

**Figure 1:**
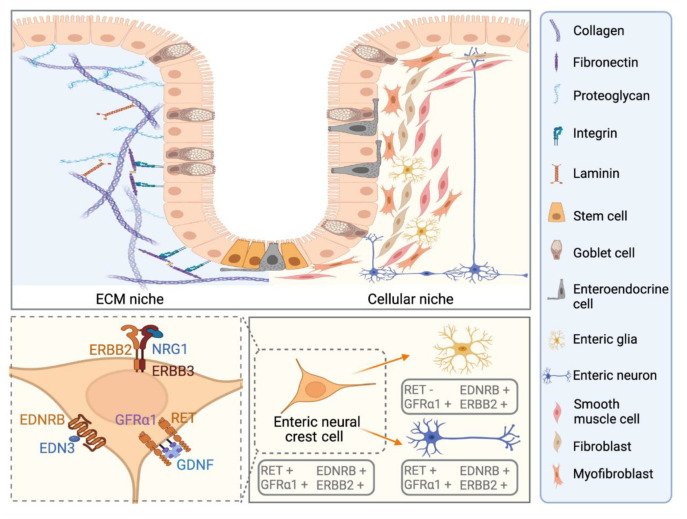
Schematic diagram of enteric neural stem cell niche in human colon. The enteric neural stem cell niche comprises the physical extracellular matrix (ECM) niche and the cellular niche located beneath the epithelial cells (upper panel). The colonic epithelium is a monlayer of polarized columnar cells (including enterocytes, goblet cells, enteroendocrine cells, and intestinal stem cells) organized as repetitive invaginations known as crypts. The ECM niche is a highly structured scaffold that provides physical and biochemical support to the surrounding cells. ECM components contain both the cell surface integrins and secreted proteins from resident cells, such as collagen, fibronectin, laminin, and proteoglycan. The resident cellular niche involves myofibroblast, fibroblast, smooth muscle cells, enteric neurons, and glia. Enteric neurons and glia are differentiated from the ENCCs. GDNF, EDN3, and NRG1 are the key niche factors that are associated with the genetic etiology of Hirschsprung disease. Together with their receptors expressed in ENCCs, including RET, EDNRB, and ERBB2/ERBB3, they form a chemoattractant gradient to mediate the proliferation, migration, and differentiation of the ENCCs (created with BioRender.com).

Vagal NCC represents the major source of ENS precursors with different subsets contributing to different regions of the bowel [15]. The cell fate of the migratory vagal NCCs is highly influenced by their interplay with the surrounding physical and the cellular niche (Figure 1; upper panel). The physical niche refers to extracellular matrix (ECM)-based scaffold that provides support to the three-dimensional structure of the bowel along which the ENCCs migrate. The cellular niche refers to the stromal microenvironment that comprises the mesenchymal cells, smooth muscle cells, endothelial cells, immune cells, and ENS cells cushioned within the ECM. These cells secrete various ECM components and niche factors which provide the essential signalling cues to direct the migrating ENCCs. Extensive studies have primarily established the contribution of two key neurotrophic factors, glial cell line-derived neurotrophic factor (GDNF) and endothelin 3 (EDN3 or ET-3), to the chemotaxis of ENCCs (Figure 1; lower panel). Emerging novel niche factors regulating ENS development, such as neuregulins and semaphorins, continue to be unveiled through genetic screening and expression profiling of HSCR patients and mutant mice models.

### 3.1. Established Roles and the Emerging Therapeutic Potentials of GDNF and EDN3 in Modulating RET and EDNRB Signalling

In humans, commencing from gestation week 4, vagal NCCs delaminate and emigrate from the neural tube, where they start to express SRY-box 10 (SOX10) and endothelin receptor B (EDNRB) [16]. Upon invading the anterior foregut, vagal NCCs express receptor tyrosine kinase (RET) [17]. Synergistically, the foregut mesenchyme expresses GDNF that preferentially binds to GDNF family receptor α1 (GFRα1) and activates RET signalling cascade. Upon activation, RET mediates phosphorylation at multiple tyrosine residues and the phosphorylated tyrosine residues serve as docking sites for various intracellular adaptor proteins, such as SRC, SHC, growth factor receptor-bound protein 2 (GRB2), phospholipase C gamma (PLCγ), to activate MAPK, RAS–ERK, phosphoinositide 3-kinase (PI3K)/Akt, and PKC pathways that influence the proliferation, migration, and differentiation of the ENCCs. Meanwhile, EDN3 (ligand of EDNRB) is expressed at high level in the midgut and hindgut, forming a gradient of chemoattractant to promote the rostro-caudal migration together with GDNF. This highly synchronized ligand-receptor expression highlights the importance of the dynamic interaction between the ENCCs and the cellular niche in defining the migratory routes and behaviours. Binding of EDN3 to EDNRB activates similar signalling pathways as RET, such as PI3K/Akt and PKC. Details of the downstream signalling cascades have been summarized in recent reviews related to ENS development [18,19,20]. In addition to modulating migration, GDNF also promotes proliferation and induces neurogenesis [21,22]. ENS precursors in mice lacking GDNF signalling during midgestation were shown to remain in an undifferentiated state, whereas recovery of the GDNF signalling restored the neuronal differentiation [22]. The potential of GDNF in regenerative medicine for HSCR was further demonstrated in that the postnatal administration of GDNF ameliorated megacolon symptoms and significantly prolonged the mean survival times of three out of four genetically distinct mouse models of HSCR [23]. The morphology and function of the GDNF-treated aganglionic colon in these mutant mice were shown to be significantly improved. Most strikingly, formation of new neurons could be induced in the cultured explants of the resected aganglionic colon of young patients with HSCR through ex vivo GDNF treatment, highlighting its immense potential in clinical application of either drug-based rectal therapy or as an adjunct treatment to optimize stem cell-based therapy. Though promising, more experimental studies are needed to fully exploit the clinical utility, efficacy, and safety of the GDNF-based treatment.

Compared to GDNF, the therapeutic potential of EDN3 remains uncertain. First, the ability of EDN3 to promote proliferation is context dependent and varies in accordance to interaction with other niche factors [24,25,26,27]. In general, various studies showed that EDN3 potentiated the proliferative effect of GDNF both in vitro and in vivo. Second, in contrast to its important role in coordinating migration of the ENCCs, EDN3 modulated the differentiation capacity of GDNF antagonistically and inhibits lineage commitment to maintain ENCCs in undifferentiated progenitor state [26,27,28]. Such negative regulation on neurogenesis, however, reduces its clinical value in therapeutic intervention for HSCR. On the other hand, EDN3 treatment to fetal mouse and avian gut explants stimulated ENCC adhesion to various ECM-associated molecules, including the ECM receptor β1-integrin [29]. The addition of EDN3 also increased the number and size of focal adhesions (FA) in which smaller FA was previously reported to associate with defects in ENS development [11]. The role of EDN3 in regulating ECM niche was further supported by the niche abnormalities observed in *Edn3^ls/ls^* (lethal spotting) mutant mice [30]. In parallel to the defective innervation of the hindgut causing distal aganglionosis, *Edn3^ls/ls^* mutant mice also exhibited upregulation of ECM components (laminins (α1, β1, and γ1) and collagens IV) in the non-neuronal mesenchymal cells of the fetal colon, resembling the ECM abnormalities reported in HSCR patients (see details in Section 3.1). Such a defect was not observed in the colon of another mouse model (*c-ret*^−/−^) for HSCR, implying that the observed ECM defect is a consequence of the underlying genetic mutation inactivating *Edn3*. These findings provide the mechanistic insights for modulating cell-ECM interaction during ENS development, implicating the potential of EDN3 in rescuing the intrinsic niche anomalies in HSCR patients.

Although null mutations of *Ret, Gdnf*, and *Gfrα1* result in nearly equivalent total colonic aganglionic phenotypes in various mice models, the phenotypic consequences and penetrance of mutations of these genes in humans are highly variable [6]. *RET* is the major gene of HSCR. High penetrant, dominant mutations of *RET* can be found in up to 50% of familial and mostly L-HSCR/TCA cases; however, for the majority of isolated and sporadic HSCR patients, damaging mutations of *RET*, except those causing loss of function (e.g., stopgain, frameshift, and splicing mutations), have incomplete penetrance. Unlike *RET*, damaging *GDNF* mutations are mostly non-Mendelian, low penetrant, and were rarely found in HSCR patients, irrespective of disease subtypes [31,32,33]. For example, in a Dutch multigenerational family with history of HSCR, additional rare variants in two Hedgehog pathway genes (*IHH* and *GLI3*) might be necessary to modify the penetrance of *GDNF* mutation for the clinical presentation of HSCR [34]. Similarly, apart from those damaging mutations causing Waardenburg syndrome, the syndromic form of HSCR characterized by pigmentation defect and deafness, *EDN3* is not frequently mutated (<3%) in isolated HSCR cases [31,33]. The reduced penetrance of *GDNF* and *EDN3* mutations implies that other niche factors may modify their regulatory effects on ENS development and may compromise the efficacy of the proposed treatment. Understanding the functional interaction of GDNF and EDN3 with other niche factors is crucial in elucidating the pathogenesis of HSCR and for the development of new alternative therapies to improve the ENS deficits.

### 3.2. Neuregulins and Semaphorins as Novel Niche Factors for ENS Dysfunction in HSCR

Amongst the niche factors with reported interaction with GDNF, neuregulins and semaphorins are putative new candidates for adjunct treatment and are frequently dysregulated in patients with HSCR. The significant contributions of common single nucleotide polymorphisms (SNPs) of neuregulin 1 (*NRG1*) and semaphorin 3C or D (*SEMA3C/D*) to HSCR were first implicated from genome wide association studies (GWAS) and the strong associations, though with small effect on disease risk, were subsequently replicated across populations [35,36,37,38,39,40,41]. In addition, damaging protein-altering mutations in *NRG1*, *NRG3,* and the receptors encoded by *ERBB2* and *ERBB3* were identified in patients with HSCR and chronic intestinal pseudo-obstruction [42,43,44]. Functionally, Nrg1 was shown to inhibit Gdnf-induced neurogenesis, while Gdnf negatively regulated Nrg1-signaling by down-regulating the expression of *ErbB2* [45]. While *NRG1* is originally known as glial growth factor (GGF), it is postulated that the antagonistic effect between *GDNF* and *NRG1* is important in maintaining the balance between neurogenesis and gliogenesis during ENS development. Specially, *NRG1* is a key axonal mediator that drives the growth, survival, and differentiation of Schwann cells and their precursors (SCP) by activating a number of downstream canonical signalling cascades, including focal adhesion kinase (FAK) [46,47]. SCP-derived neurons constitute a significant part of the ENS, and it was reported that up to 20% of the myenteric and submucosal neurons of the postnatal colon are from Schwann cell lineage [15,48]. As demonstrated in mouse models, SCP-derived neurogenesis can occur in a *Ret*-independent manner [49]. This finding suggests that SCP can be a cellular source to restore ENS function in HSCR patients with defective RET signalling, and the migration of SCP can be further directed by NRG1 treatment. Recent studies using single cell RNA-sequencing on intestinal niche further uncovered the role of mesenchymal-derived NRG1 as a potent promoter of stem cell identity and mediator of tissue regeneration [50]. NRG1 was upregulated in mesenchymal stromal cells upon damage and exogenous NRG1 treatment can promote in vitro intestinal organoid formation and proliferation following injury [51]. NRG1 could also support enteroid growth and enhanced cell type diversity. To summarize, NRG1 may act as an important niche factor in the prospective SCP-based cell therapy and help other translational research that use colonic organoid to mimic the in vivo gut environment for disease modelling.

Semaphorins belong to a class of membrane-bound or secreted proteins that bind to plexins and/or neuropilins as co-receptors to regulate cellular responses, including cell migration and cell–cell communication. Specifically, class 3 semaphorins are secreted proteins associated with ENS development and pathogenesis of HSCR. Although both common regulatory and rare coding variants in *SEMA3C/D* were reported to be associated with HSCR, their contributions to the genetic etiology seem to be limited to European populations [36,40,52]. Functional characterization using knockdown of *sema3c* and *sema3d* in zebrafish embryos showed reduction in migratory ENS precursors and the co-knockdown with *ret* displayed synergistic effects of complete ablation of gut innervation. An expression profiling study on intestine of *Ret^k−/k−^* mutant mice revealed downregulation of collapsin response mediator family members, *Crmp1* and *Dpysl3/Crmp4*, involved in mediating the intracellular Sema3A signalling [53]; however, the therapeutic potential of semaphorin remains unexplored thus far.

### 3.3. Other Niche Factors Implicated by Genetic Defects in HSCR Patients

Other transcription and growth factors with genetic defects in HSCR, e.g., *SOX10* and *PHOX2B* underlying syndromic forms of HSCR and fibroblast growth factors (*FGFs*) disrupted in protein interaction network associated with S-HSCR, may also help optimize the in vitro culture conditions for cell-based therapy [6,54]. In addition, the retinoic acid receptor gamma (*RARG*) and its downstream targets were also found to be perturbed in S-HSCR [54]. Retinoic acid (RA) signalling is essential for normal ENS development. Vitamin A deficiency in *Rbp^–/–^* mutant mouse model caused distal bowel aganglionosis and cell-autonomous loss of RA receptor signalling affected ENCC migration and differentiation in a stage-specific and context-dependent manner [55,56]. Retinoic acid is therefore important not only in ENS precursor induction and specification in cell-based therapy, but also in modulating migration and neurogenesis after transplantation.

## 4. ECM Niche in ENS Development

Apart from the cellular niche factors, abnormal physical niche of the gut often underlies HSCR [3,57]. The physical niche is the non-cellular three-dimensional ultrastructure of ECM and functional molecules secreted by resident cells, which links together growth factors and other bioactive molecules to control diverse cellular functions, including innervation, stem cell differentiation, and cell adhesion. ECM molecules include structural proteins, such as collagen and laminin that are crosslinked to provide the structural integrity to the intestinal epithelium, as well as binding sites for other ECM molecules, such as fibronectin and proteoglycans. Through an integrated set of sensor and effector circuits involving multiple ligands and receptors, ECM niche provides instructive signals and orchestrates cellular behaviour essential for ENS development. Intriguingly, the composition and organization of the ECM changes dynamically in response to the surrounding cellular microenvironment and in turn influences its regulation on cellular functions. Thus, dysregulation of ECM niche is not only a consequence, but can also be a driver of the pathogenesis of HSCR. Recently, genetic and functional studies unveiled additional HSCR-associated niche defects. Harnessing these niche abnormalities to modulate the ECM can be a novel complementary strategy to promote regeneration of the ENS in the distal colons of HSCR patients.

### 4.1. Established Findings of ECM Abnormalities along the Colon of HSCR Patients

Interactions between the ENCCs and ECM play a pivotal role in the formation of the ENS. In the 1990s, abnormal gene expression and distribution of ECM molecules were already reported in HSCR colons. Increased abundance of the ECM components and the associated adhesion proteins, including collagen type IV, laminin, tenascin, and fibronectin, were observed in smooth muscle layers of normoganglionic bowel specimens of HSCR patients than the colon of the healthy controls [58,59,60]. More recently, the serum level of fibronectin was found to be higher in patients. Even along the HSCR colon, abnormal distribution of fibronectin and collagens (I and III) was also reported, with higher expression detected in the aganglionic segment, but the level decreased in the transitional zone and normoganglionic bowel [61,62,63]. These findings suggested that altered composition of the ECM correlates with abnormal innervation, implicating that the absence of enteric neurons may have additional adverse effects on the ECM niche. Interestingly, bulk RNA sequencing of ENCCs derived from patient-specific iPSCs showed perturbation of pathways related to morphogenesis of the epithelium and ECM organization, calling into question whether the observed ECM aberrations are non-cell autonomous or cell autonomous effects of the ENCCs [31].

### 4.2. Recent Findings of ECM Abnormalities in Colon of Mutant Model Organisms

Similar observations of ECM abnormalities have been reported in the context of mutant model organisms. As mentioned before, anomalies in ECM, including overabundance of laminin, tenascin, and collagen type IV, were detected in the distal colons of *Edn3^ls/ls^* and *Ednrb^flex3/flex3^* mice showing incomplete colonization [64,65]. Such changes in ECM were postulated to delay the migration of ENCCs, leading to premature neuronal differentiation and hence distal aganglionosis. The importance of cell-ECM interaction was further demonstrated in a β1-integrin^–/–^ mouse model in which the conditional ablation of *Itgb1* in ENCCs resulted in impaired adhesion on ECM, migratory defects, and colonic aganglionosis [66]. Integrins are heterodimeric transmembrane receptors, each composed of one α and one β subunit. Different combinations define the ligand-binding specificities to ECM molecules, such as collagen, laminin, and Arg-Gly Asp (RGD) receptors [67]. For example, integrins α2β1 and α5β1 bind to collagen I and fibronectin, respectively, whereas integrin α7β1 binds to laminin 2 [68]. They mediate cell–cell adhesion or cell-ECM interaction via multiple signalling pathways (e.g., FA regulation) and transduce signals that regulate gene expression and cell growth. Not only does integrin signalling contribute to cell autonomous effect on migration, endothelial cells can arrest ENCC migration through inhibiting cell-ECM interaction between β1 integrin on the surface of ENCCs and ECM proteins expressed by the intestinal vasculature [69]. Additionally, other studies demonstrated that ENCCs could autonomously remodel their niche microenvironment to promote their migration through spatiotemporally production of tenascin-C (TNC), collagens (Col6a4 and Col18), and agrin [70,71,72]. Both positive and negative effects of TNC on cell adhesion and migration have been reported depending on the cell types, species, and context of analysis. In an avian model, TNC was expressed by ENCCs at the wavefront to actively promote migration [70]. It had an anti-adhesive effect to inhibit integrin-mediated cell adhesion to fibronectin, and thereby allowed faster detachment of ENCCs from the ECM to facilitate their own migration; however, TNC was reported to inhibit migration in mouse gut explant cultures [73]. The species-specific effect may possibly be due to the differences in the expression pattern of TNC in distal colon across species. On the contrary, agrin is not expressed in undifferentiated ENCCs at the wavefront and, consistent with its later expression during ENS development, it was shown to strongly inhibit ENCC migration. While *Col18* is permissive to ENCC migration, collagen VI can interfere with the promigratory effect of fibronectin by downregulating focal adhesion proteins, and acts as a poor substratum in supporting ENCC migration [74]. Altogether, these findings highlight the cell-autonomous role of the ENCCs in active remodelling of the local microenvironment. In parallel, the ECM niche also influences the stem cell fate of the migrating ENCCs in a non-cell-autonomous manner. While ECM components represent appealing therapeutic targets, further investigation into the link between the ECM and ENCCs can provide an additional level of control over the stem cell fate and regulation.

### 4.3. Novel Discoveries of Intrinsic Niche Defects of HSCR Patients Attributed to Genetic Aberrations from Recent Sequencing Studies

The advent of high throughput next generation sequencing (NGS) has greatly accelerated the pace of discovery of novel genes associated with congenital disorders in genome-wide scale. In the case of HSCR, NGS-based DNA sequencing permits unbiased genome/exome-wide genetic profiling irrespective of prior research focus on the ENCC lineage. The unbiased approach unequivocally demonstrated the presence of intrinsic niche defects attributed to rare mutations in ECM-associated genes across disease subtypes of HSCR [31,75].

**Figure 2:**
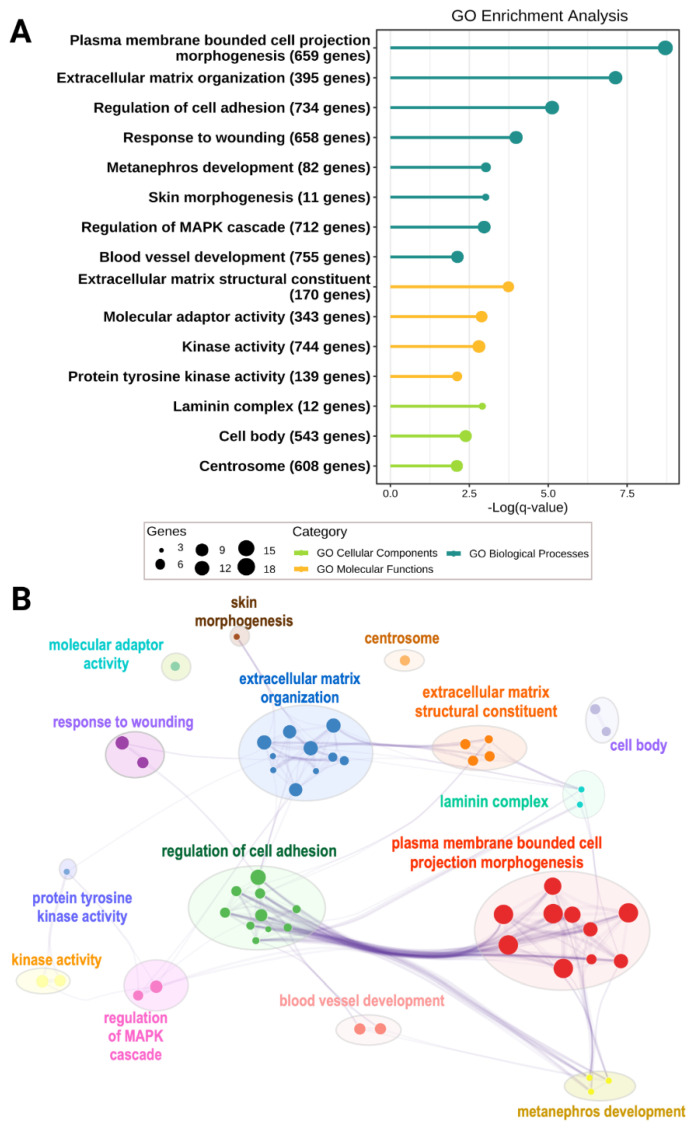
Gene ontology (GO) enrichment of rare de novo, biallelic, and digenic genotypes in L-HSCR patients (Data from Tang et al. [75]). (**A**) Top GO terms enriched in genes (*n* = 77) with rare de novo or biallelic genotypes identified from L-HSCR patients’ functional analysis of variants (*q*-value < 0.01). Among the 77 genes, 58 genes have either de novo or biallelic variants, and 19 genes encode interacting partners to any of these 58 candidate genes. (**B**) Interaction network of GO enriched terms grouped by functional relevance using Metascape. Top GO cluster of plasma membrane bounded cell projection morphogenesis represents GO terms related to cell morphogenesis involved in neuron differentiation, neuron projection guidance and axon guidance. Other closely interacted GO clusters included regulation of cell adhesion, ECM organization, and structural constitute, as well as laminin complex. Each node represents a GO term, and only GO terms with *q*-value < 0.01 are shown.

Significant functional enrichment of rare de novo, biallelic, or digenic genotypes, mainly in the ECM-related pathways, involving ECM organization and structural components (including laminin and collagen) as well as cell adhesion (including integrins), was discovered in a family based whole genome sequencing (WGS) study of nine sporadic L-HSCR/TCA cases devoid of damaging mutations in major HSCR genes (Figure 2). For some patients with L-HSCR, a network of interacting ECM/ECM-associated proteins (e.g., collagens (COL6A2; COL6A3; and COL1A2), laminins (LAMB2 and LAMA5), and integrins (ITGA2 and ITGB5)) and niche factors (NRG1) were disrupted by rare protein-altering alleles inherited from different parents [75]. These findings corroborate the ECM abnormalities reported in the colon of patients, and suggested that HSCR patients may have intrinsic niche defects dysregulating ENS development.

**Figure 3:**
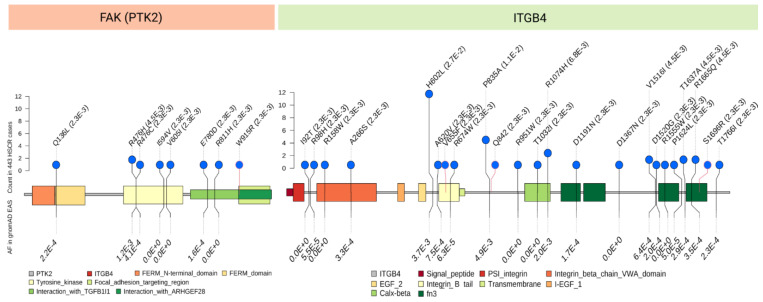
Mutation spectrum of the two genes (*FAK*/*PTK2* and *ITGB4*) with increased burden of rare damaging variants in S-HSCR cases (Data from Tang et al. [31]). Count of the variant is represented by the height of the lines with the blue dot, while the minor allele frequency is represented by the annotation in brackets following the protein mutation label on the top. The colour of the dot border represents whether this mutation is reported in the gnomAD database (black is reported and orange is novel) and the frequency in gnomAD East Asia is labelled at the bottom.

Later, a population-based WGS study on 443 S-HSCR patients further detected enrichment of rare damaging variants in two genes encoding the ERBB2-interacting partners—integrin beta 4 (*ITGB4*) and *FAK*—in HSCR patients through interactome analysis [31]. FAK lies downstream of NRG1 activation in Schwann cells, and is critical in promoting proliferation of the Schwann cells and preventing them from premature differentiation [47,76]. Interestingly, FAK also directly interacts with RET, which might provide the functional convergence between the two seemingly unrelated core HSCR pathways [77]. The distribution of mutations appears different between the two genes, with damaging mutations scattering across the tyrosine kinase and FA targeting domains for *FAK*, whereas the association of *ITGB4* is context-dependent (Figure 3). *ITGB4* encodes the integrin beta 4 subunit, a receptor for the laminins, and tends to form heterodimer with alpha 6 subunit. The increased mutational burden of *ITGB4* in HSCR cases is largely attributed to a single missense mutation encoding p.His602Leu near the EGF domain, which might have a specific effect on protein structure and function; however, the functional role of *ITGB4* in ENS development remains largely unexplored.

**Figure 4:**
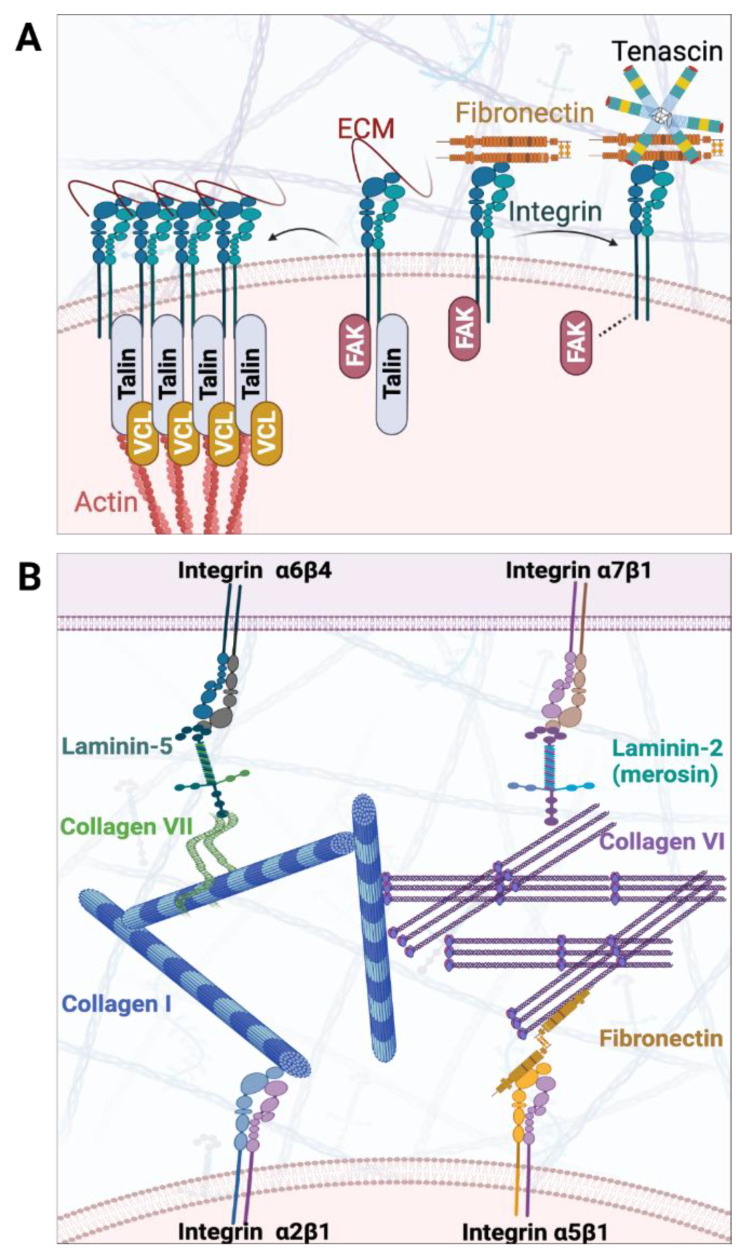
Schematic diagram of ECM components dysregulating ENS development in HSCR from genetic studies. (**A**) Focal adhesion kinase (FAK) involves in the intracellular signalling cascade initiated by activation of integrins. FAK promotes the recruitment of talin, and vinculin (VCL) can then bind to integrin-bound talin to crosslink actin to regulate the signal transduction from the ECM to the actin cytoskeleton. Integrins with RGD domain can bind fibronectin and the binding of tenascin inhibits fibronectin-induced integrin signalling. (**B**) Impaired Cell-ECM interaction in HSCR patients involving collagens, laminins, and integrins. Adhesion to collagen I can be mediated by integrin α2β1. Integrin α5β1 can attach to collagen VI via fibronectin. Laminin, which play as a major component of the basal lamina, can promote cell motility. Cross-talk between collagens and laminins is found in collagen VI and laminin-2, as well as collagen VII and laminin-5 (created with BioRender.com).

In fact, integrins are tightly linked to FA. FA are large molecular assemblies that link the cytoskeleton of a cell to the ECM via integrin receptors and adaptor proteins such as talin and vinculin (*VCL*) (Figure 4A). A key regulator of FA is FAK, which regulates the stabilization and turnover of FA complexes not only for cell adhesion but also for regulatory signal transduction. Integrins with RGD domain can bind with fibronectin and the induced integrin signalling can be inhibited by tenascin partly through modulating FAK. FAK also promotes the recruitment of talin. *VCL* can then bind to the integrin-bound talin and crosslink talin with actin for FA assembly. The genetic contribution of FA to pathogenesis of HSCR was first highlighted through the discovery of association of *VCL* to S-HSCR in an integrative genomic study involving whole exome sequencing of HSCR patient and RNA sequencing of the ENCCs differentiated from patient-specific iPSCs [11]. The ENCCs derived from iPSCs of HSCR patients with *VCL* mutation recapitulated the HSCR-like phenotype of delayed migration and defective formation of neuronal axons, alongside reduction in FA size. Encouragingly, genome correction of the *VCL* mutation in patient-specific iPSCs with CRISPR/Cas9-mediated targeting approach restored the FA size and hence rescued the differentiation and migration defects of the derived ENCCs. This functional characterization of patient-derived ENCCs and subsequent rescue experiment demonstrated that abnormal FA and impaired cell-ECM interaction may directly interfere with the cellular behaviour of ENCCs and their responses to the niche microenvironment, thereby resulting in defects in differentiation and migration of the ENCCs. To summarize, these NGS findings support the long-standing hypothesis of the intrinsic niche defects in HSCR patients. Genetic mutations may underlie such niche defects, impairing the cell-ECM interaction and resulting in defective ENS development (Figure 4B). Deciphering the pathogenic mechanisms of how the genetic mutations in HSCR patients give rise to niche defects is crucial to restore the normal niche or ameliorate their niche defects to safeguard the success of the clinical application of stem cell-based therapy.

## 5. Conclusions and Future Perspectives on Regenerative Medicine of HSCR

Enteric neural stem cell niche is the dynamic microenvironment along which the ENCCs migrate during embryogenesis. Critical to the development of the ENS are the niche factors acting as microenvironmental cues as well as the cell–cell adhesion molecules and ECM that controls niche functions and organization. Both the cellular niche factors, such as GDNF, EDN3, and NRG1, and ECM are essential components of the niche underlying the genetic etiology of HSCR. Genetic defects disrupting the functions of these critical components may interfere with the regulation of migration, proliferation, and differentiation of both the endogenous and exogenous ENCCs, which can reduce the effectiveness of stem cell-based therapies (Table 1). Identification and subsequent rescue of these genetically regulated niche defects by targeting the abnormal niche therapeutically in patients might reverse the impaired cellular behaviour of the transplanted ENCCs. This intervention includes the administration of small molecules targeting the defective niche factors or cell-ECM interactions to facilitate the engraftment and integration of the enteric neuronal progenitors. Ultimately, an optimal treatment for HSCR may involve combinations of molecules targeting the ENS precursors and the niche, applied at different times and at different locations to restore the dynamics of cell–niche interactions in the aganglionic colon.

Exceptional progress in NGS technology has dramatically reduced the cost of NGS, allowing for comprehensive genomic and transcriptomic profiling of HSCR patients and animal models of HSCR. In particular, single-cell RNA sequencing (scRNA-seq) and spatial transcriptomics have offered a new dimension for studying the transcriptional landscape of fetal gut of human and mouse models at single cell spatial resolution. Determining the differential patterns of (i) transcriptional landscape of cell types populating the fetal colon and (ii) expression profiles corresponding to distinct anatomical regions or developmental stages in diseased versus normal gut samples would help decipher the spatiotemporal coordination of interactions between the niche and ENCCs. Improved understanding of the cell–niche interaction holds great promise in maintaining the optimal microenvironment for the patterning and development of the exogenous ENCCs in regenerative cell-based therapy.

Recent advances in regenerative medicine offer new innovative solutions to ENS dysfunction. ECM represents an ideal therapeutic substrate for functional remodelling of the aganglionic bowel tissues. The elucidation of the compositional and structural changes in ECM and the abnormal cell-ECM interaction in patients will pave the path towards gut tissue engineering. Using ECM bioscaffold for the anatomically precise reconstruction of a functional intestine provides the platform to support and transplant the allogenic or autologous enteric neural stem cells, which maybe a long-term goal to treat severe HSCR patients.

## Figures and Tables

**Table 1:** Genes reported with common HSCR-associated variants or rare damaging protein-altering variants in HSCR patients.

Gene	Phenotype	Mode of Inheritance ^a^	Variant Spectrum
Cellular niche factors			
*GDNF*	Isolated HSCR	AD	Rare
*EDN3*	Isolated HSCR	AR/AD	Rare
	Waardenburg syndrome	AD	Rare
*ECE1*	HSCR with cardiac, craniofacial, and autonomic defects	AD	Rare
*SOX10*	Isolated HSCR	AD	Rare
	Waardenburg syndrome	AD	Rare
*PHOX2B*	Congenital Central Hypoventilation Syndrome with HSCR	AD	Rare
	Isolated HSCR	Additive	Common
*NRG1*	Isolated HSCR	AD and additive	Rare and common
*SEMA3C/D*	Isolated HSCR	AD and additive	Rare and common
ECM niche factors			
*COL6A2*	Isolated HSCR	AD	Rare
*COL6A3*	Isolated HSCR	AD	Rare
*COL1A2*	Isolated HSCR	AD	Rare
*LAMB2*	Isolated HSCR	AD	Rare
*LAMA5*	Isolated HSCR	AD	Rare
*ITGA2*	Isolated HSCR	AD	Rare
*ITGB4*	Isolated HSCR	AD	Rare and low frequency
*ITGB5*	Isolated HSCR	AD	Rare
*PTK2/FAK*	Isolated HSCR	AD	Rare
*VCL*	Isolated HSCR	AD	Rare

^a^ AD: autosomal dominant; AR: autosomal recessive.

## Data Availability

Not applicable.
